# Feasibility of Prehabilitation Prior to Breast Cancer Surgery: A Mixed-Methods Study

**DOI:** 10.3389/fonc.2020.571091

**Published:** 2020-09-25

**Authors:** Priya Brahmbhatt, Catherine M. Sabiston, Christian Lopez, Eugene Chang, Jack Goodman, Jennifer Jones, David McCready, Ian Randall, Sarah Rotstein, Daniel Santa Mina

**Affiliations:** ^1^Faculty of Kinesiology and Physical Education, University of Toronto, Toronto, ON, Canada; ^2^Department of Supportive Care, Princess Margaret Cancer Centre, Toronto, ON, Canada; ^3^Faculty of Medicine, University of Toronto, Toronto, ON, Canada; ^4^Department of Surgical Oncology, Princess Margaret Cancer Centre, Toronto, ON, Canada; ^5^Department of Anesthesia and Pain Management, University Health Network, Toronto, ON, Canada

**Keywords:** prehabilitation, breast cancer, survivorship, rehabilitation, oncology, surgery

## Abstract

**Background:**

Breast cancer surgery results in numerous acute and long-term adverse outcomes; the degree to which these can be mitigated or prevented through prehabilitation is unknown.

**Methods:**

We conducted a longitudinal, single-arm, mixed-methods study to examine the feasibility of prehabilitation in 22 women undergoing breast cancer surgery. All participants received an individualized exercise prescription including upper quadrant-specific resistance and mobility training and aerobic exercise for the duration of their surgical wait time. Feasibility was assessed by recruitment, adherence, attrition, and intervention-related adverse event rates. An exploratory investigation of intervention efficacy was conducted via a 6-min walk test, upper-quadrant strength and range of motion, volumetric chances associated with lymphedema, and participant-reported quality of life, fatigue, pain, and disability. Outcome assessments were conducted at baseline, prior to surgery, and at six and 12 weeks after surgery. Semi-structured interviews with a subset of participants (*n* = 5) and health-care providers (H; *n* = 2) were conducted to provide further insights about intervention feasibility. Qualitative data were analyzed using a hybrid inductive and deductive thematic analysis approach.

**Results:**

Recruitment and attrition rates were 62 and 36%, respectively. Average prehabilitation duration was 31 days (range = 7–69 days). Seventy six percent of participants complied with at least 70% of their prehabilitation prescription. There was a clinically significant increase in the 6-min walk distance from baseline to the preoperative assessment (57 m, 95% CI = −7.52, 121.7). The interviews revealed that the intervention was favorably received by participants and HCPs and included suggestions that prehabilitation (i) should be offered to all surgical candidates, (ii) is an avenue to regain control in the preoperative period, (iii) is a facilitator of postoperative recovery, and (iv) is an opportunity to provide education regarding postoperative rehabilitation protocols. A preference for multimodal prehabilitation (including dietetic and psychological counseling) was also highlighted.

**Conclusion:**

Our findings suggest that surgical prehabilitation in women with breast cancer is feasible. Data are hampered by study sample size and lack of a control group. Thus, randomized controlled trials to examine prehabilitation efficacy in people with breast cancer, especially interventions employing a multimodal strategy, are warranted.

## Introduction

Breast cancer is the most common malignancy and principal cancer-related cause of death in adult females in industrialized nations (1). Surgery is a cornerstone of therapy and is indicated in more than 90% of people with breast cancer at some point during treatment (2). While highly effective at disease control, it often results in physical and psychosocial sequelae that significantly impair quality of life and may last for months or years after treatment completion (3, 4). For example, common regional postoperative effects include lymphedema, pain, axillary web syndrome, and upper-quadrant dysfunction, which manifests as a loss of strength and range of motion in chest, shoulder, arm, and cervical spine (5–8). Furthermore, whole-body adverse effects such as fatigue, which is disproportionately higher in people with breast cancer compared to other cancer populations, (9, 10) is reported by up to 95% of all patients during therapy (9). The severity of these symptoms, however, varies depending on a number of factors, including age, comorbid conditions, treatment regimen, and baseline physical well-being (11, 12). Higher levels of preoperative aerobic fitness are associated with better surgical outcomes including decreased postoperative complications and mortality in other clinical (13) and cancer populations (14–16). Although the relationship between objectively measured physical fitness and surgical outcomes in individuals with breast cancer has not been elucidated, higher physical activity levels are associated with earlier postoperative recovery (17). Taken together, this evidence suggests that physical fitness is a modifiable risk factor that may be targeted to improve surgical outcomes.

A burgeoning body of research is investigating the utility of preoperative interventions, known as prehabilitation, to optimize posttreatment health outcomes. Numerous reviews of the prehabilitation literature in cancer populations demonstrate several important benefits, including improved preoperative and postoperative physical function, reduced hospital length of stay, and fewer postoperative complications (18–24). However, this literature exists almost exclusively in people undergoing tumor resection for thoracoabdominal malignancies, with breast cancer prehabilitation remaining largely unexamined. In the only breast cancer surgery prehabilitation study to date, Baima and colleagues (25) found that teaching preoperative shoulder stretches for individuals undergoing breast cancer surgery was feasible via in-person or by video with similar postoperative outcomes across groups. The feasibility and effects of prehabilitation targeted at improving broader markers of quality of life and symptom burden, such as fitness, fatigue, and pain before and after surgery, are otherwise unknown. As a preliminary step at furthering this field of research, we sought to assess the feasibility and acceptability of an individualized, home-based prehabilitation intervention prior to breast cancer surgery using a mixed-methods approach. The secondary objective was to explore the potential benefit of prehabilitation on physical fitness and participant-reported physical and psychosocial well-being over time to inform future studies with point estimates and variability data.

## Materials and Methods

### Study Design

This study was a prospective, single-arm, feasibility study with an emergent, embedded mixed-methods design. Qualitative methodology was implemented part-way through the study to further understand the participants’ experience with prehabilitation and their preferences regarding intervention design. This study was approved by the University Health Network Research Ethics Board (#16-6165), and all participants provided written informed consent prior to initiating any study activity.

### Sampling and Eligibility

A convenience sample of people undergoing breast cancer surgery was recruited from breast cancer clinics at the Princess Margaret Cancer Centre. Participants were eligible if they (i) were diagnosed with stage I–III breast cancer; (ii) consented to surgery (mastectomy or lumpectomy); (iii) had a surgical waiting period of at least 3 weeks; (iv) were proficient in English; or (v) were between the ages of 18 and 80 years. Patients were excluded from the trial if they (i) received or were receiving neoadjuvant treatment; (ii) had medical contraindications to exercise; or (iii) had active shoulder pathology. Qualitative interview participants were recruited via convenience sampling from the quantitative strand (i.e., individuals who had participated in prehabilitation). In addition to conducting semi-structured interviews among patient-participants, we recruited health-care practitioners (HCPs) from the breast cancer clinic via convenience sampling to provide their perceptions regarding the feasibility and value of prehabilitation for people with breast cancer.

### Intervention

The prehabilitation intervention comprised of individually tailored, home-based exercise prescriptions commencing immediately following the baseline assessment and until the day of surgery. The exercise prescriptions were developed and delivered by a Registered Kinesiologist (RKin) and consisted of aerobic exercise 3 to 5 days per week for 30–40 min per session, and upper quadrant-specific resistance training 2 to 3 days per week. Aerobic exercise prescriptions typically included brisk walking at an intensity of four to six on a 10-point rating of perceived exertion (RPE) scale (26). Upper quadrant-specific resistance training consisted of two to three sets of 10 to 12 repetitions per exercise, with each session incorporating up to eight exercises (standing rows, shoulder external rotation, front raise, lateral raise, bicep curls, triceps extensions, wall push-ups, and chest press). Training progression per modality was guided by the RKin and occurred when the participant could complete the aerobic exercise with mild exertion (RPE of 0–3) or when the participant could complete 15 repetitions of any of the resistance exercises without eliciting at least moderate exertion (3–6) on the RPE scale. The intervention also included stretching and mobility exercises which reflected standard postoperative rehabilitation. This allowed participants to familiarize themselves with postoperative protocols while functionally unimpaired.

All participants were provided with resistance bands and an exercise manual to facilitate home-based exercise. The RKin communicated with the participants on a weekly basis via phone calls or emails to support program compliance and appropriate progression and to address any barriers to exercise (including questions about appropriate exercise completion) that may have prevented ongoing participation.

### Outcomes

Demographic, disease, and treatment-related data were collected at baseline from the participant and by chart review. Measures of intervention efficacy were collected at baseline, approximately 1 week prior to surgery and at 6 and 12 weeks postoperatively. Qualitative interviews with patient-participants were conducted at the last study assessment or shortly thereafter. Qualitative interviews with HCPs were conducted after all participants had completed the intervention.

#### Quantitative Feasibility Outcomes

The recruitment rate was calculated as the number of participants successfully consented over the total number of patients approached. Intervention adherence was captured through participant self-report via exercise logs. Adherence to resistance training was calculated as the volume of exercise repetitions completed relative to the lower end of the range of repetitions prescribed. Adherence to the aerobic exercise was defined as total quantity completed per week relative to the lower end of the range prescribed. Attrition was assessed as the number of participant-withdrawals relative to the participants who consented and was reported per assessment timepoint. Reasons for participant withdrawal were also collected. Intervention-related adverse event information was collected from the participants during weekly communication with the RKin. Lastly, participant satisfaction was collected at the last study assessment via a study-specific satisfaction questionnaire.

*A priori*, we determined that feasibility would be confirmed with (i) a recruitment rate >60%; (ii) >70% intervention adherence; (iii) attrition rate <30%; and (iv) no serious adverse events [defined as anything above a Grade 2 of the CTCAE v5 (27)] related to participation in the prehabilitation intervention. Participant satisfaction was captured through a satisfaction survey to understand the participant’s experience with the intervention.

#### Quantitative Exploratory Outcomes

Aerobic functional capacity was measured using the 6-min walk test (6MWT) (28). Upper-extremity strength was measured via handgrip dynamometry (Jamar^®^, Chicago, IL, United States) and manual muscle testing using a digital handheld dynamometer (MicroFET2; Hoggan Scientific^®^, Salt Lake City, UT, United States) for elbow flexion and extension, and shoulder abduction, flexion, and extension. An active range of motion of the glenohumeral and scapulothoracic joints was measured via goniometry for the following actions: shoulder flexion, extension, internal and external rotation, and abduction. Other measurements included waist circumference (WC), body mass index (BMI), lean body mass, body fat percentage (BF%), and fat mass. Upper-extremity limb size to detect potential development of lymphedema was measured via circumferential measurements at (i) the metacarpophalangeal joints; (ii) the wrist; (iii) 10 cm distal to the lateral epicondyles; and (iv) 15 cm proximal to the lateral epicondyles (29).

Participant-reported upper-quadrant function was collected using the disabilities of the arm, shoulder and hand (DASH) questionnaire. The brief pain inventory (BPI) was used to collect cancer-specific pain (30). Fatigue was assessed using the fatigue subscale of the Functional Assessment of Cancer Therapy-Fatigue (FACT-F) questionnaire (31). Health-related quality of life (HRQOL) was measured using the second version of the 36-Item Short Form Health Survey (SF-36 v2) (32). The Godin–Shephard Leisure Time Exercise Questionnaire-Leisure Score Index (GLTEQ-LSI) was used to measure physical activity levels (33, 34). Lastly, global level of functioning and disability were measured using the 36-item World Health Organization Disability Assessment Schedule 2.0 (WHODAS 2.0) (35).

#### Qualitative Assessment of Feasibility and Participant Experience

The purpose of the participant interviews was to understand their experience with prehabilitation and different factors that affected feasibility of the intervention (e.g., challenges to participation and preferences regarding the exercise prescription and intervention delivery). We sought to interview all participants to understand the variability in individual experiences because of the different life stages and physical activity backgrounds of the participants. To further understand intervention design and viability, as well as the perceived value of prehabilitation, we also interviewed HCPs within the breast cancer clinic. All interviews were semi-structured and included open-ended questions along with relevant prompts. The interview guide was pilot tested to allow the interviewer to ensure familiarity with the script. All interviews were conducted either in-person or over the telephone by the RKin, were recorded, and were transcribed verbatim prior to analysis.

### Data Analysis

#### Quantitative Data

Participant demographics and clinical characteristics were analyzed using descriptive statistics [mean ± standard deviation, and frequency (%)]. Participation rates, reasons for exclusion and dropout, and attrition rates were analyzed by reason frequency and percentages as appropriate. Adherence to the prehabilitation prescription was expressed as a percentage of exercise completed relative to the minimum training volume prescribed for both aerobic and resistance training. Participants were also categorized as adherent (completed >70% of their exercise prescription in each session), partially adherent (completed <70% of their exercise prescription in some sessions), or non-adherent (completed <70% of their prescription in all sessions). Descriptive statistics were also used to analyze the frequency of responses in the participation satisfaction survey.

Exploratory outcomes were assessed using a linear mixed-effects model to assess changes over time. Models were fitted with the following variables as fixed effects: (i) Surgery type (categorized into either lumpectomy or mastectomy); (ii) Measurement timepoint; and (iii) Prehabilitation duration (in number of days). Individual participants were included as random effects. Comparisons between timepoints were made using Tukey HSD (honest significant differences) *post hoc* pairwise comparisons, and data were analyzed under the intention-to-treat principle. Missing data values were accounted for using maximum likelihood estimation (with the assumption that data are missing at random) in the model. All analyses were done in R version 3.4.1.

#### Qualitative Data

Interview data were analyzed using Braun and Clarke’s six-step approach for thematic analysis in a hybrid deductive and inductive manner (36, 37). A deductive analysis approach allowed for a detailed examination of themes directly related to the interview questions. For data that emerged during the interview but was not planned or directly related to the interview questions, we used an inductive analysis approach. This approach is data-driven and allows for the development codes and themes based on the content of the data, rather than trying to strictly fit the data into a preexisting framework or theory (36). Interview transcripts were read multiple times by the first author, and emerging concepts were identified via memoing. Codes were then categorized into themes. Descriptions of the themes were created, and representative quotes were chosen and reviewed by the last author.

#### Integration of Quantitative and Qualitative Data

There were multiple points of quantitative and qualitative integration in this study. Integration at the methods level was achieved through the sampling frame where participants for the qualitative portion were recruited from those who had participated in the quantitative portion (38). Mixing of the methods also occurred at the interpretation and reporting phase, where quantitative and qualitative data are integrated using a narrative weaving approach and reported together on a theme-by-theme basis (38).

## Results

### Quantitative Feasibility Findings

**FIGURE 1:**
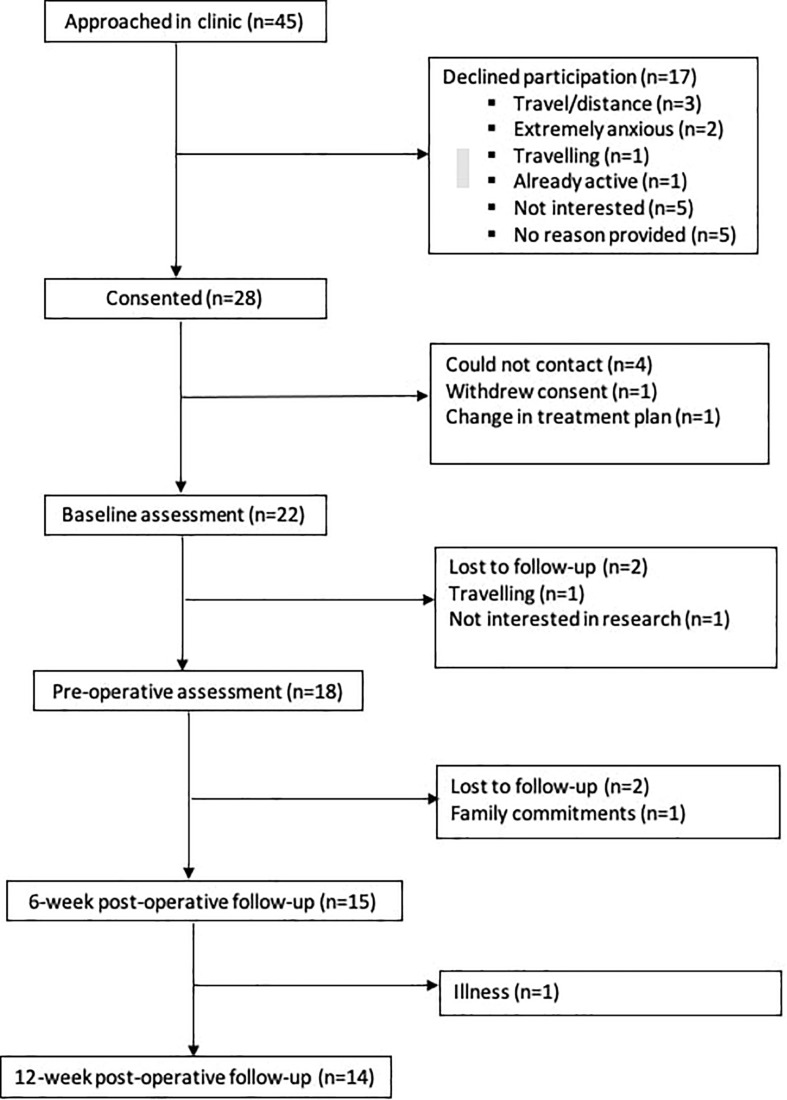
Study flow diagram.

The study flow diagram is presented in Figure 1, and participant demographics are in Table 1. From April 2017 to July 2018, 45 eligible patients were approached in clinic, of whom 28 (62%) consented to participate in the study. Primary reasons for declining participation were travel/distance-related concerns (*n* = 3), too much anxiety to commit to prehabilitation (*n* = 2), and lack of interest in exercise/research participation (*n* = 5). Five patients did not provide a reason for declining participation. Twenty-two (*n* = 22) participants attended the baseline assessment and received the intervention. Reasons for dropout between study consent and the baseline assessment included change in treatment plan resulting in ineligibility (*n* = 1) and withdrawal from the study due to time constraints (*n* = 1). The study team was unable to contact four participants to book study visits. Study enrollment rate, calculated as number of participants who received the intervention relative to the number approached, was approximately 49%. The overall attrition rate from baseline to the last study assessment was approximately 36% (*n* = 8). The average prehabilitation window (i.e., the period from the baseline to preoperative assessment) was approximately 30 ± 16.59 days. The surgical wait time for individuals in this study (i.e., the date from treatment decision to the date of surgery) was 38 ± 16.56 days. There were no intervention-related adverse events during the study.

**TABLE 1:** Participant baseline characteristics (*n* = 22).

Characteristic	Mean ± SD

Age	54.18 (±10.98)

	Frequency (%)
**Ethnicity**	
White/Caucasian	14 (63.64)
Latino/Hispanic	2 (9.09)
East Asian	2 (9.09)
South East Asian	1 (0.05)
South Asian	1 (4.55)
Ashkenazi Jewish	1 (4.55)
Prefer not to answer	1 (4.55)
**Marital status**	
Married	12 (54.55)
Divorced	2 (9.09)
Single	2 (9.09)
Common-law	2 (9.09)
Widowed	1 (4.55)
Other	2 (9.09)
Prefer not to answer	1 (4.55)
**Education**	
Finished University/college	15 (68.18)
Some University/college	3 (13.64)
Some high school	1 (4.55)
Other	2 (9.09)
Prefer not to answer	1 (4.55)
**Working status**	
Working/studying full-time	11 (50.00)
Working/studying part-time	2 (9.09)
Retired	2 (9.09)
Unemployed	1 (4.55)
Disability/sick leave	2 (9.09)
Other	3 (13.64)
Prefer not to answer	1 (4.55)
**Socioeconomic status**	
>$75,000	9 (40.91)
40,000–$75,000	2 (9.09)
20,000–$39,000	2 (9.09)
<$20,000	3 (13.64)
Prefer not to answer	6 (27.27)
**Surgery type**	
Unilateral lumpectomy with SLNB	12 (54.55)
Unilateral mastectomy with SLNB	2 (9.09)
Unilateral mastectomy with ALND	1 (4.55)
Bilateral mastectomy with SLNB	4 (18.18)
Bilateral mastectomy with SLNB and insertion of tissue expanders	1 (4.55)
Bilateral mastectomy with immediate autologous reconstruction	1 (4.55)

Five (23%) participants did not submit their exercise logs for adherence analysis. On average, adherence to the minimum range of the aerobic exercise prescription was 142.22 ± 82.66% and adherence to the resistance training prescription was 114.44 ± 38.26%. Adherence levels exceeded 100% because most participants were exercising beyond the lower end of their exercise prescription range. Of the 17 participants that provided adherence data, 13 (76%) were considered adherent to their prescription (i.e., completed >70% of exercise volume prescribed for each session). Two participants partially adhered to their prescription (i.e., completed <70% of their prescribed exercise volume in some sessions), and two participants were non-adherent (i.e., completed <70% of their prescribed exercise volume in all the sessions).

Eleven participants completed the participant satisfaction survey at the final study assessment. Of those, all 11 (100%) reported that they experienced benefits from participating in the study, had no side effects or harm related to the study, and did not consider discontinuing participation. Ten (90.9%) found the exercise manual helpful, and eight (72.7%) said they were able to complete all the exercises prescribed to them. On average, participants rated the program 8.6 out of a score of 10, with 0 being the lowest and 10 being the highest score possible. All respondents indicated that they planned to continue exercising on a regular basis (45–60 min per day, 3–4 days per week), would recommend the program to anyone else undergoing surgery, and believed that prehabilitation helped them recover after surgery.

### Quantitative Exploratory Findings

Mean scores for objectively measured outcomes of physical fitness and participant-reported outcome measures are presented in Tables 2, 3, respectively. Between-timepoint differences for physical fitness and participant-reported outcomes have been reported in Supplementary Tables 1, 2. Because the primary purpose of this study was not to assess intervention efficacy, the sample was underpowered to detect statistically significant differences in exploratory outcomes. As such, we have highlighted outcomes, which demonstrated clinically meaningful changes.

**TABLE 2:** Mean estimates ± SE (95% CI) for objectively measured physical fitness outcomes (*n* = 22).

Outcome	Baseline	Preoperative	6-week postoperative assessment	12-week postoperative assessment
6MWT (m)	474 ± 19.9 (433, 514)	531 ± 22.6 (485, 576)	525 ± 24.2 (476, 574)	536 ± 22.6 (491, 582)
Weight (kg)	77.5 ± 3.23 (70.7, 84.4)	77.7 ± 3.24 (70.8, 84.6)	77.5 ± 3.25 (70.6, 84.4)	77.9 ± 3.24 (71.0, 84.8)
Waist circumference (cm)	94.7 ± 2.22 (90.0, 99.4)	96.6 ± 2.28 (91.8, 101.4)	96.5 ± 2.35 (91.6, 101.4)	97.7 ± 2.30 (92.9, 102.5)
Body fat (%)	38.0 ± 1.73 (34.4, 41.6)	38.0 ± 1.83 (34.2, 41.8)	37.6 ± 1.94 (33.6, 41.6)	36.4 ± 1.86 (32.6, 40.2)
BMI (kg/m^2^)	29.7 ± 1.26 (27.1, 32.4)	29.7 ± 1.27 (27.1, 32.4)	29.6 ± 1.27 (26.9, 32.2)	29.8 ± 1.27 (27.1, 32.5)
Hand grip strength (kg)	51.6 ± 3.17 (45.1, 58.1)	51 ± 3.41 (44.0, 57.9)	50.0 ± 3.69 (42.5, 57.4)	52.8 ± 5.50 (45.7, 59.9)
**Upper-extremity strength (kg)**				
Elbow flexion	22.5 ± 1.28	22.8 ± 1.34	20.69 ± 1.60	20.9 ± 1.43
Elbow extension	22.2 ± 1.38	21.6 ± 1.43	19.9 ± 1.62	19.5 ± 1.49
Shoulder flexion	18.3 ± 0.94	18.1 ± 1.02	17.2 ± 1.30	17.8 ± 1.09
Shoulder extension	25.6 ± 1.75	27.2 ± 1.84	26.5 ± 2.38	24.8 ± 1.98
Shoulder abduction	16.7 ± 0.92	16.8 ± 1.01	15.2 ± 1.36	15.7 ± 1.10
**Shoulder range of motion (°)**				
Right flexion	161 ± 3.98 (153, 169)	165 ± 4.38 (156, 174)	141 ± 4.82 (131, 151)	149 ± 4.52 (140, 158)
Right extension	60.2 ± 3.43 (53.1, 67.3)	61.6 ± 3.56 (54.3, 68.9)	56.0 ± 3.76 (48.3, 63.7)	61.3 ± 3.63 (53.8, 68.8)
Right abduction	160 ± 4.99 (150, 170)	167 ± 5.52 (156, 178)	147 ± 6.11 (135, 159)	156 ± 5.70 (145, 168)
Right internal Rotation	46.6 ± 3.34 (39.8, 53.4)	53.6 ± 3.83 (45.9, 61.3)	45.0 ± 4.32 (36.3, 53.7)	52.5 ± 4.14 (44.2, 60.9)
Right external rotation	97.3 ± 4.46 (88.0, 107.0)	100.3 ± 4.81 (90.4, 110.0)	93.9 ± 5.16 (83.4, 104.0)	90.7 ± 4.91 (80.7, 101.0)
Left flexion	163 ± 4.00 (155, 172)	163 ± 4.28 (155, 172)	158 ± 4.47 (149, 167)	161 ± 4.30 (152, 170)
Left extension	56.5 ± 3.43 (49.4, 63.5)	57.5 ± 3.65 (50.0, 65.0)	57.8 ± 3.83 (50.0, 65.5)	60.2 ± 3.66 (52.7, 67.7)
Left abduction	163 ± 4.52 (154, 173)	163 ± 4.84 (153, 173)	159 ± 5.05 (149, 170)	165 ± 4.85 (155, 175)
Left internal rotation	46.9 ± 3.62 (39.4, 54.3)	51.5 ± 3.97 (43.5, 59.6)	43.8 ± 4.19 (35.3, 52.3)	54.5 ± 3.98 (46.4, 62.6)
Left external rotation	91.0 ± 6.20 (77.9, 104.2)	93.5 ± 6.34 (80.2, 106.8)	89.0 ± 6.42 (75.6, 102.5)	85.2 ± 6.34 (71.8, 98.5)
**Lymphedema (cm)**				
Right MCP joints	20.1 ± 0.29 (19.5, 20.7)	19.6 ± 0.30 (19.0, 20.2)	19.7 ± 0.31 (19.1, 20.4)	19.6 ± 0.30 (19.0, 20.3)
Right wrist	16.6 ± 0.27 (16.0, 17.1)	16.4 ± 0.28 (15.8, 16.9)	16.5 ± 0.29 (15.9, 17.1)	16.5 ± 0.28 (15.9, 17.0)
Right 10 cm distal to lateral epicondyles	25.2 ± 0.57 (24.0, 26.4)	25.2 ± 0.58 (24.0, 26.4)	25.2 ± 0.59 (24.0, 26.4)	25.0 ± 0.58 (23.8, 26.2)
Right 15 cm proximal from lateral epicondyles	33.8 ± 1.04 (31.6, 36.0)	33.1 ± 1.07 (30.9, 35.3)	33.3 ± 1.08 (31.1, 35.6)	33.5 ± 1.07 (31.3, 35.7)
Left MCP joints	19.6 ± 0.27 (19.0, 20.2)	19.7 ± 0.28 (19.2, 20.3)	19.6 ± 0.29 (19.0, 20.2)	19.7 ± 0.28 (19.1, 20.3)
Left wrist	16.3 ± 0.23 (15.9, 16.8)	16.2 ± 0.24 (15.7, 16.7)	16.2 ± 0.24 (15.7, 16.7)	16.4 ± 0.24 (15.9, 16.9)
Left 10 cm distal to lateral epicondyles	25.1 ± 0.54 (23.9, 26.2)	24.9 ± 0.55 (23.7, 26.1)	25.2 ± 0.56 (24.0, 26.4)	25.0 ± 0.55 (23.8, 26.1)
Left 15 cm proximal from lateral epicondyles	33.4 ± 1.02 (31.1, 35.5)	33.0 ± 1.04 (30.8, 35.1)	32.9 ± 1.05 (30.7, 35.1)	33.1 ± 1.04 (30.9, 35.3)

*6MWT, 6-min walk test; MCP, metacarpophalangeal; Hand grip strength and upper-extremity strength values are reported as a sum of both sides.*

**TABLE 3:** Mean estimates ± SE (95% CI) for participant-reported outcomes (*n* = 22).

Outcome	Baseline	Preoperative	6-weeks postoperative assessment	12-weeks postoperative assessment
GLTEQ	22.8 ± 5.30 (11.91, 33.7)	37.9 ± 6.10 (25.53, 50.3)	21.7 ± 6.31 (8.92, 34.5)	33.8 ± 6.12 (21.38, 46.2)
WHODAS: Average Disability Score	30.0 ± 2.34 (25.1, 34.8)	29.2 ± 2.57 (23.9, 34.5)	33.2 ± 2.69 (27.7, 38.7)	30.2 ± 2.63 (24.8, 35.5)
FACT-F	37.9 ± 2.54 (32.7, 43.1)	39.7 ± 3.02 (33.6, 45.8)	36.0 ± 3.26 (29.4, 42.6)	33.3 ± 3.13 (26.9, 39.6)
BPI: Severity	1.63 ± 0.36 (0.89, 2.36)	2.03 ± 0.40 (1.21, 2.84)	1.72 ± 0.42 (0.86, 2.58)	1.82 ± 0.41 (0.98, 2.66)
BPI: Interference	1.33 ± 0.48 (0.34, 2.33)	1.48 ± 0.53 (0.39, 2.58)	2.18 ± 0.56 (1.04, 3.32)	1.54 ± 0.55 (0.42, 2.65)
DASH	8.99 ± 3.54 (1.73, 16.2)	11.97 ± 4.28 (3.31, 20.6)	28.16 ± 4.44 (19.19, 37.1)	20.96 ± 4.28 (12.28, 29.6)
SF-36: PCS	35.50 ± 1.46 (32.6, 38.5)	33.70 ± 1.79 (30.1, 37.4)	30.50 ± 1.95 (26.6, 34.5)	29.60 ± 1.86 (25.9, 33.4)
SF-36: MCS	43.00 ± 1.70 (39.5, 46.5)	41.20 ± 1.96 (37.2, 45.2)	45.60 ± 2.09 (41.3, 49.8)	44.90 ± 2.02 (40.8, 49.0)

*GLTEQ, Godin Leisure Time Exercise Questionnaire; WHODAS, World Health Organization Disability Assessment Schedule; FACT-F, functional assessment of cancer therapy—fatigue; BPI, brief pain inventory; DASH, disabilities of arm, shoulder, and hand; SF-36 PCS, 36 item short form survey physical component score; SF-36 MCS, 36 item short form survey mental component score.*

The 6-min walk distance increased from baseline to the preoperative assessment by 57.10 ± 24.0 m (95% CI = −7.52, 121.7). While there was a small decrease in 6MWT distance from the preoperative assessment to the 6-week postoperative assessment (−5.51 ± 27.6 m [−79.74, 68.7]), scores remained greater than at baseline. There was an overall increase in 6MWT distance of 62.90 ± 24.00 m (−1.81, 127.60) from baseline to the last study assessment. Although a minimal clinically important difference (MCID) has not been established in the breast cancer setting, in other cancer populations it has been identified to be around 20 m (39). The overall 6MWT distance change represents almost three times the MCID. All other physical fitness outcomes remained relatively stable over the study period.

An increase in DASH scores of 16.18 ± 4.96 (2.74, 29.63) points was observed between the preoperative and 6-week postoperative assessment, indicating a clinically important increase in upper-quadrant disability (MCID of 15 points) (40). From baseline to the 12-week postoperative assessment, there was an overall worsening in fatigue levels demonstrated by a reduction of 4.63 ± 3.34 (−13.7, 4.41) points in FACT-F scores which have an MCID of three points (41). The physical component score of the SF-36 questionnaire consistently worsened over the study period with a decrease of 5.90 ± 2.17 (−11.75, −0.05) points from the first to the last assessment. The mental component score, on the other hand, worsened from baseline to the preoperative assessment but then improved by 4.36 ± 2.25 (−1.72, 10.44) points from the pre- to 6-week postoperative assessment. The MCID for SF-36 scores is between 3 and 5 points in various clinical populations (42). Lastly, GLTEQ-LSI scores increased over the study period from 22.8 ± 5.30 at baseline to 33.8 ± 6.12 at the last study assessment, which reflects a change from being insufficiently active at baseline according to physical activity guidelines for cancer survivors to being sufficiently active at 12 weeks after surgery (43, 44).

### Qualitative Findings

Five participants and two HCPs who are both clinical nurse coordinators volunteered to participate in the interviews. A total of eight themes emerged, which were then grouped into two distinct categories (intervention feasibility and participant experience) described below. Representative quotes for each theme are provided in Table 4.

**TABLE 4:** Selected quotes from participant and health care provider interviews.

Theme Subtheme	Representative quote(s)
**Intervention Feasibility**	
Appropriateness of the intervention	“So I followed the exercises [prescribed as part of the prehabilitation intervention] in addition to the ones I was already going to do and I mean they were easy exercises…anyone could have done them, which made it really great for any women of any age at any physical level.” [ID01]
	“Those [resistance bands] were fun! I’ve never used them before, and they were easy to use. I actually went away for a few days during the period before the surgery and it was great because I could stick them in a suitcase.” [ID28]
Barriers and facilitators to participation	***Barriers:*** “Weather would dictate walking. I was doing some rebounding back then as well, so there were no barriers there in my house…so other than weather for getting outside, nothing.” [ID20] ***Facilitators:*** “What I found most helpful was doing a run through with you of them [exercises] the first time out, making sure you’re doing them correctly.” [ID24] “That checklist [exercise log] recommended how many times a week to do each activity and it gave me that motivation to check that off and I brought that back to you. It held me accountable to keep doing the exercise…” [ID16]
Target population	“I actually have said to many nurses and doctors after that it [prehabilitation] should be something that is mandatory and should be implemented at the hospital for every person going through the surgery.” [ID24]
	“I think it’s valuable for everyone. I just think it’s such an overwhelming time for patients, so when we present it as an option, I totally get why some women aren’t interested but I think if you present it as this valuable resource, it’s going to be really helpful for you recovery.” [HCP1]
**Participants’ Experiences**	
Intervention design preferences	
Multimodal care	“Those things [diet, psychological wellbeing] do go hand-in-hand. You know, the diet and exercise are key things around making a successful recovery, so if there was a way to engage that…that might be helpful to other patients as well.” [ID24]
Need for an exercise professional	“When you’re having very specific surgery, where someone is actually familiar with it, it’s not like I can call up a physio center and say, ‘oh can you help me because these problems.’ You need to have someone who specializes and recognizes what the issues are…” [ID24].
	“I think it would be a good idea to do it [exercise training] in person maybe, but I’m not sure I would have been able to make every appointment…I think it might be a nice idea but I think you would find some scheduling challenges for people.” [ID28]
Perceived benefit	“…she actually did DIEP breast reconstruction, which is a huge surgery. By the next day she was able to lift her arms over her head, which for that population of patients, that takes months to do after surgery. So, she had said it was really because of the prehabilitation.” [HCP2]
	“I really feel like I benefited a lot from it because it caught me in that time just after diagnosis when things were pretty scary and pretty awful and I felt like it was one of the key pieces of my plan for positivity during this whole thing, because it was setting a tone for recovery.” [ID20]
Health behavior change	“I feel like being in programs like this [post-treatment rehabilitation], which started with the prehab, kept a momentum going. And, I’m not completely on my own yet, but it’s great to have these kinds of programs at my disposal and it’s helping me to stay active. I think prehab was the one to get that rolling.” [ID16]
Regaining control	“I think for just the average person, it [prehabilitation] shares a lot of knowledge and I think knowledge is power and it gives someone the ability to take things into their own hands, where a lot of their control and power is being taken away from them.” [ID01]
Prehabilitation as education	“You get some limited guidance from the surgeon and nurse about stretching and mobility exercises after the surgery, but it’s not a lot. There was one group class and it’s at a time when I probably wasn’t that focused – the day before surgery – wasn’t really focused on exercises. So, I thought it was helpful that I had the contact with you because it helped me figure out what I should do and that I should keep going.” [ID28]

#### Intervention Feasibility

Elements related to feasibility of the intervention were coded and categorized into the following three themes: (i) Appropriateness of the intervention, (ii) Barriers and facilitators to participation, and (iii) Target population.

The appropriateness of the intervention was discussed by the women. Participants described the prehabilitation intervention as convenient because the prescription is entirely bodyweight- and resistance band-based. Some participants traveled during the preoperative period and were able to continue exercising because of the portability of the resistance bands. Further, participants described the intervention as easy to follow; individualization of the prescription allowed each participant to receive a program that they were able to follow with ease regardless of previous physical activity experience.

While the intervention was deemed appropriate, there were both barriers and facilitators to participation. It was evident through the interviews that both participants and HCPs recognized that there might be challenges to optimal uptake of the intervention. Potential barriers that emerged were related to motivation and the weather. Lack of time was another important barrier that was commonly referred to by participants because the preoperative period is typically occupied with many medical appointments and personal/professional responsibilities. While those were the only barriers mentioned, a couple of characteristics of the intervention design surfaced as potential facilitators of exercise intervention adherence. Participants reported that the in-person instruction of the exercises, which allowed them to practice and receive feedback, was especially helpful and increased how comfortable participants felt with being able to exercise on their own at home. Moreover, the weekly phone conversations along with the exercise logs, which were given to participants to track adherence, also appeared to be important facilitators to exercise adherence. Participants said that they created a sense of accountability, which was further augmented by the structure of the exercise prescription.

Based on the discussions pertaining to the intervention, it was recognized that not all patients would be willing to participate in prehabilitation; generally, both the participants and the HCPs suggested that prehabilitation should be made available to everyone receiving surgery.

#### Participant Experiences

Concepts related to the participants’ experiences with prehabilitation were collated into the following themes: (i) Intervention design preferences; (ii) Perceived benefit; (iii) Health behavior change; (iv) Regaining control; and (v) Prehabilitation as education.

Participants shared their preferences regarding the prehabilitation intervention including what they enjoyed and what they would have liked to see as part of the study. These preferences were further described and organized into the subthemes of (i) “multimodal care” which explains the need to include other complementary modalities of health behavior change in the prehabilitation intervention and (ii) “the need for an exercise professional” which highlights the need for an exercise professional to be delivering prehabilitation and rehabilitation-related programming. Firstly, participants almost unanimously spoke about the need to include either dietetic and/or a psychological support to help with stress and emotion management to optimize health in the preoperative period, while recognizing that there may be differing individual needs and preferences. Secondly, participants indicated that having an oncology-trained exercise professional was an asset that allowed them to feel more comfortable with their prehabilitation regimens. They recognized that having a trained professional would provide insight that might not be available if they were to seek out exercise support independently. Whether participants wanted consistent supervised training from the exercise professional was mixed. While some suggested that it might be helpful to have weekly sessions, others said that the home-based prescription was more appropriate given time constraints.

All the participants reported experiencing benefit from prehabilitation, including the perception that it facilitated earlier recovery and provided a positive distraction. In addition to the specific benefits from the intervention, many participants identified prehabilitation as a catalyst for positive health behavior changes. They reported that prehabilitation provided the momentum to make health behavior changes that they had been intending to make not just in the preoperative period but also in the postoperative period. Furthermore, many participants reported that prehabilitation allowed them to regain a sense of control during an otherwise tumultuous period where individuals often felt stripped of their autonomy. In this way, the loss of control that was frequently discussed as a result of frequent medical visits and impending treatment was partially addressed with a prehabilitation program.

Finally, the use of the prehabilitation intervention prior to surgery as a tool to educate patients also emerged as an aspect of the intervention that participants found to be helpful. It allowed participants the opportunity to ask questions that may not have the chance to ask their oncologists/nurses given the time constraints during their medical appointments and to learn about what they *should* be doing rather than what they *should not*.

## Discussion

Our primary objective was to assess the feasibility and acceptability of a home-based prehabilitation intervention prior to breast cancer surgery. We were able to recruit 28 patients out of the 45 that were approached (62%), slightly surpassing our anticipated recruitment rate of 60% to indicate feasibility for future studies. Study enrollment rate was approximately 49%. However, the attrition rate at the last study timepoint was 36% which was higher than the pre-decided threshold for success (30% overall attrition). Reasons for dropout included illness, other time commitments, and travel. These have previously been cited in the literature as barriers to participation in clinical trials, including exercise studies (45–47). The perioperative period may be especially susceptible to attrition given the substantial burden associated with medical visits at that time. The attrition rate in this study was slightly higher than the 25% reported by Baima and colleagues in their breast cancer surgical prehabilitation study (25). The difference in attrition rates between the two studies may be attributed to the fact that Baima and colleagues collected data at the follow-up oncology appointments and did not require additional center visits. Extra hospital visits, as well as illness and treatment-related mood disturbances, have previously been cited as a reason for dropout from clinical trials (45–48).

Overall adherence to the intervention in this study was impressive, with most participants exercising more than they were prescribed (approximately 142 and 114% for aerobic and resistance exercise, respectively), with over 75% of participants of those who provided data adhering fully to their prescription. There were no adverse events related to the intervention. Collectively, these results suggest that the exercise intervention that was used in this study is both safe and feasible for this population. This is unsurprising given that patients are typically asymptomatic and are not functionally limited prior to surgery compared to the acute postoperative/adjuvant treatment period. In fact, the preoperative window may be when patients are at their healthiest during their treatment course. Qualitative findings were congruent with the quantitative adherence data. Only a few participants reported experiencing any barriers to participation (e.g., weather, motivation, and time) but usually would find an alternative exercise modality, which would allow them to adhere to their prescription. Instead, participants found that elements of the program (e.g., a home-based setting, using resistance bands, having to report adherence) facilitated adherence to the protocol, which explains the high adherence rates. Baima and colleagues (25) found that 76% of their participants chose to exercise and of these, 85% exercised on three or more days per week. A recent review of prehabilitation prior to intra-abdominal cancer surgery reported that home-based trials had adherence rates of approximately 70% whereas supervised trials reported adherence rates of about 98% (49).

The participant satisfaction survey and qualitative findings from the interviews suggest that individuals had an extremely positive experience with prehabilitation. According to the satisfaction survey, all participants indicated that they (i) benefited from being in the study; (ii) felt like prehabilitation facilitated recovery after surgery; (iii) would recommend prehabilitation to anyone else with breast cancer who underwent surgery; and (iv) intended to continue exercising regularly (45–60 min per day, 3–4 days per week). These different perceptions of benefit also emerged in all of the participant interviews. Participants said that they felt like prehabilitation expedited their postoperative recovery and that they felt better during subsequent treatment(s) because of it. Although participants were not prescribed any postoperative exercise through the study, many indicated that they continued exercising during adjuvant therapy. For many participants in this study, participation in the prehabilitation program provided an opportunity to discuss safety concerns related to exercising during adjuvant treatment.

An important theme that was identified in the interviews was the need for a multimodal intervention, including exercise, dietetic support, and stress management counseling delivered by the appropriate professionals. This need for multimodal prehabilitation has been repeatedly identified in the literature (50, 51). Advocacy from researchers and clinicians alike has resulted in a shift toward multimodal interventions in research and practice given that these different modalities likely were synergistically and provide greater benefit than either modality alone might (52, 53). In this study, participants highlighted the need for an exercise professional to be delivering information related to prehabilitation and rehabilitation, given that their oncology care providers may be unable to provide adequate information. A recent study by Nadler and colleagues (54) found that close to 80% of oncology care providers were unaware of cancer exercise guidelines for survivors and recognized a lack of knowledge, time, and concerns regarding safety as barriers to conversations surrounding exercise. As such, these health-care providers recognized the need for an exercise specialist to be included as part of the clinical team.

Prehabilitation as a catalyst for positive health behavior change also emerged as a prevalent theme expressed by participants in the qualitative investigation. Some participants reported that prehabilitation provided the momentum to make changes in health behaviors (e.g., exercise behaviors and diet habits) that they had been intending to make. Quantitative findings reflected these reports as seen by the changes in GLTEQ-LSI scores. These data suggest that there was an increase in self-reported physical activity between baseline and the preoperative assessment; at baseline, average GLTEQ-LSI scores for the study sample represented them as being insufficiently active [not meeting physical activity guidelines (55)].

While these are early findings which need confirmation via adequately powered randomized controlled trials, a few outcomes demonstrated clinically meaningful changes over the study period. Most notably, functional aerobic capacity scores increased well beyond clinically important margins from baseline to the preoperative assessment. There was a small decrease in 6MWT scores at the 6-week postsurgery assessment and subsequent increase in scores at the final study assessment. Importantly, scores did not return to baseline after surgery. Contemporary prehabilitation trials have largely included supervised exercise prescriptions. From those that have utilized home-based prescriptions similar to this study, increases in 6MWT scores in the preoperative period range from 25 to 42 meters over an average duration of around 30 days (53, 56, 57). While there are no normative values established for the 6MWT in this population, other trials of home-based exercise in the *posttreatment* setting have reported an average change of 60 m after a 12-week intervention with baseline values of approximately 417 m and post-intervention values of 477 m (58). The greater improvements in 6MWT scores in the present study may be explained by the high adherence rates compared to the aforementioned studies.

Self-reported disability was collected using the WHODAS 2.0 and DASH questionnaires which assess global and upper quadrant-specific disability, respectively. Scores from both measures reflected the greatest disability at the 6-week postoperative assessment. Changes in DASH scores from presurgery to postsurgery suggested a clinically important change in disability, which improved but did not return to baseline at the 12-week postoperative assessment. While there is substantial data supporting the use of physiotherapy after surgery to facilitate shoulder function recovery, (59–61) no studies to date have implemented this type of protocol preoperatively. Qualitative findings demonstrated that participants in this study continued to exercise after surgery, as reflected by the quantitative GLTEQ-LSI data. Presumably, this may have facilitated recovery of shoulder function as some participants stated in their interviews; however, it is difficult to ascertain this without a control group.

Health-related quality of life worsened slightly over the study period, as measured by the SF-36 questionnaire. These findings are in line with those from a review of surgical prehabilitation in a heterogeneous patient group which found that preoperative exercise interventions do not significantly affect HRQOL after surgery (62). Some data suggest that psychological prehabilitation might be beneficial in maintaining HRQOL before and after treatment (63). Taken together, these findings imply that a multimodal prehabilitation approach might be more helpful to address perioperative HRQOL and well-being, as suggested in the literature (50) and in the qualitative findings of the present study. Fatigue improved between baseline and the preoperative assessment but progressively worsened thereafter. The decline in scores at the 6-week postoperative assessment was the largest and reflected a clinically important change in fatigue levels. Treatment-related fatigue in cancer survivors is one of the most common and debilitating side effects of treatment, (9, 64) and in women with breast cancer, pretreatment fatigue is one of the strongest predictors of persistent fatigue after treatment (65). Exercise is established as one of the most effective interventions to mitigate cancer-related fatigue (66) but has yet to be used prophylactically. Data from the present study suggest that prehabilitation may improve fatigue levels prior to surgery; as such, it may have a role in attenuating persistent fatigue given the aforementioned relationship between pretreatment and posttreatment fatigue.

This study had several strengths including the novelty of the intervention in this population, the use of mixed methodology, which allowed for a comprehensive understanding intervention feasibility and participant experiences, and the inclusion of a large breadth of exploratory outcomes, which provide pilot data for sample-size calculations for future studies. Interpretation of findings, however, must be cautioned given the single-arm design which was underpowered to detect statistically significant changes in the included outcomes. The lack of between-group comparisons with an intervention-naïve group makes it impossible to comment on intervention efficacy, but the observed clinically meaningful changes warrant further investigation. In addition to the small sample size, there was a relatively high attrition rate in this study suggesting that the follow-up timepoints may be difficult for participants to attend; this may be because individuals may be undergoing adjuvant therapy after surgery and experiencing radiation and chemotherapy-related adverse effects. Further, because participants were not reimbursed for study assessments, these visits may have been a financial burden that contributed to the high attrition rate. Other limitations include the late inclusion of qualitative interviews because of which we were unable to capture interview data from a large proportion of participants, especially those who had dropped/were not compliant; small qualitative sample which did not capture the breadth of the participants’ experiences (i.e., those who participated in the interviews were those who were compliant and enjoyed the intervention); and self-reporting of exercise adherence and physical activity levels which, while common in exercise oncology literature, are often overreported (67).

## Conclusion

Our data suggest that home-based prehabilitation prior to breast cancer surgery is feasible and favorably received by participants. For women undergoing breast cancer surgery, prehabilitation may facilitate postoperative recovery, impact health behavior change in the preoperative and postoperative periods, and improve physical activity levels and functional capacity both preoperatively and postoperatively. While these findings are encouraging and largely reflect previous prehabilitation research, adequately powered trials of multimodal prehabilitation in women with breast cancer are needed to confidently determine intervention efficacy.

## Data Availability Statement

The datasets presented in this article are not readily available because data collected cannot be shared outside of the research institution. Requests to access the datasets should be directed to corresponding author.

## Ethics Statement

The studies involving human participants were reviewed and approved by the University Health Network Research Ethics Board. The patients/participants provided their written informed consent to participate in this study.

## Author Contributions

All authors listed have made a substantial, direct and intellectual contribution to the work, and approved it for publication.

## Conflict of Interest

The authors declare that the research was conducted in the absence of any commercial or financial relationships that could be construed as a potential conflict of interest.
