# Supplementary material for: Feasibility of Prehabilitation Prior to Breast Cancer Surgery: A Mixed-Methods Study
Source: Front Oncol. 2020 Sep 25;10:571091. doi: 10.3389/fonc.2020.571091 (PMC7544900; doi:10.3389/fonc.2020.571091)
Supplement: Supplementary file 1 [file Data_Sheet_1.docx]

Supplemental Table 1: Mean Time point Differences for Objectively Measured Physical Fitness Outcomes (n=22)

| **Outcome** | **Contrast** | **Δ ± SE** | **95% CI** |
| --- | --- | --- | --- |
| **6MWT (m)** | Baseline – pre-op | 57.10 ± 24.0 | -7.52 – 121.7 |
|  | Pre-op – 6 weeks post-op | -5.51 ± 27.6 | -79.74 – 68.7 |
|  | 6 weeks post-op - 12 weeks post-op | 11.31 ± 26.9 | -61.06 – 83.7 |
|  | Baseline – 6 weeks post-op | 51.6 ± 25.60 | -17.16 – 120.30 |
|  | Baseline – 12 weeks post-op | 62.90 ± 24.00 | -1.81 – 127.60 |
|  | Pre-op – 12 weeks post-op | 5.80 ± 25.70 | -63.65 – 75.10 |
| **Weight (kg)** | Baseline – pre-op | 0.19 ± 0.68 | -1.65 – 2.03 |
|  | Pre-op – 6 weeks post-op | -0.25 ± 0.81 | -2.43 – 1.92 |
|  | 6 weeks post-op - 12 weeks post-op | 0.43 ± 0.79 | -1.68 – 2.55 |
|  | Baseline – 6 weeks post-op | -0.06 ± 0.75 | -2.08 – 1.96 |
|  | Baseline – 12 weeks post-op | 0.37 ± 0.71 | -1.53 – 2.27 |
|  | Pre-op – 12 weeks post-op | 0.18 ± 0.75 | -1.83 – 2.19 |
| **Waist circumference (cm)** | Baseline – pre-op | 1.88 ± 1.27 | -1.54 – 5.31 |
|  | Pre-op – 6 weeks post-op | -0.09 ± 1.50 | -4.13 – 3.96 |
|  | 6 weeks post-op - 12 weeks post-op | 1.24 ± 1.46 | -2.71 – 5.18 |
|  | Baseline – 6 weeks post-op | 1.80 ± 1.40 | -1.97 – 5.56 |
|  | Baseline – 12 weeks post-op | 3.03 ± 1.31 | -0.51 – 6.57 |
|  | Pre-op – 12 weeks post-op | 1.15 ± 1.39 | -2.60 – 4.89 |
| **Body Fat (%)** | Baseline – pre-op | 0.02 ± 1.44 | -3.86 – 3.90 |
|  | Pre-op – 6 weeks post-op | -0.41 ± 1.70 | -4.97 – 4.16 |
|  | 6 weeks post-op - 12 weeks post-op | -1.23 ± 1.66 | -5.69 – 3.24 |
|  | Baseline – 6 weeks post-op | -0.06 ± 0.75 | -2.08 – 1.96 |
|  | Baseline – 12 weeks post-op | 0.37 ± 0.71 | -1.53 – 2.27 |
|  | Pre-op – 12 weeks post-op | 0.18 ± 0.75 | -1.83 – 2.19 |
| **BMI (kg/m^2^)** | Baseline – pre-op | 0.01 ± 0.26 | -0.70 – 0.71 |
|  | Pre-op – 6 weeks post-op | -0.13 ± 0.31 | -0.97 – 0.70 |
|  | 6 weeks post-op - 12 weeks post-op | 0.17 ± 0.30 | -0.64 – 0.99 |
|  | Baseline – 6 weeks post-op | -0.13 ± 0.29 | -0.90 – 0.65 |
|  | Baseline – 12 weeks post-op | 0.05 ± 0.27 | -0.68 – 0.78 |
|  | Pre-op – 12 weeks post-op | 0.04 ± 0.29 | -0.73 – 0.81 |
| **Hand Grip Strength (kg)** | Baseline – pre-op | -0.58 ± 3.20 | -9.21 – 8.04 |
|  | Pre-op – 6 weeks post-op | -1.02 ± 3.77 | -11.14 – 9.10 |
|  | 6 weeks post-op - 12 weeks post-op | 2.83 ± 3.69 | -7.11 – 12.77 |
|  | Baseline – 6 weeks post-op | -1.60 ± 3.51 | -11.06 – 7.85 |
|  | Baseline – 12 weeks post-op | 1.23 ± 3.30 | -7.67 – 10.12 |
|  | Pre-op – 12 weeks post-op | 1.81 ± 3.50 | -7.60 – 11.22 |
| **Upper Extremity Strength (kg)** |  |  |  |
| Elbow flexion | Baseline – pre-op | 0.31 ± 1.45 | -3.62 – 1.23 |
|  | Pre-op – 6 weeks post-op | -2.23 ± 1.73 | -6.91 – 2.45 |
|  | 6 weeks post-op - 12 weeks post-op | 0.28 ± 1.73 | -4.40 – 4.95 |
|  | Baseline – 6 weeks post-op | -1.93 ± 1.65 | -6.38 – 2.53 |
|  | Baseline – 12 weeks post-op | -1.65 ± 1.48 | -5.67 – 2.37 |
|  | Pre-op – 12 weeks post-op | 1.95 ± 1.56 | -6.17 – 2.26 |
| Elbow extension | Baseline – pre-op | -0.55 ± 1.28 | -4.01 – 2.92 |
|  | Pre-op – 6 weeks post-op | -1.71 ± 1.53 | -5.84 – 2.43 |
|  | 6 weeks post-op - 12 weeks post-op | -0.43 ± 1.51 | -4.53 – 3.67 |
|  | Baseline – 6 weeks post-op | -2.53 ± 1.44 | -6.17 – 1.66 |
|  | Baseline – 12 weeks post-op | -2.68 ± 1.30 | -6.21 – 0.84 |
|  | Pre-op – 12 weeks post-op | -2.14 ± 1.37 | -5.86 – 1.58 |
| Shoulder flexion | Baseline – pre-op | -0.26 ± 1.14 | -3.34 – 2.82 |
|  | Pre-op – 6 weeks post-op | -0.91 ± 1.44 | -4.79 – 2.98 |
|  | 6 weeks post-op - 12 weeks post-op | 0.66 ± 1.46 | -3.29 – 4.61 |
|  | Baseline – 6 weeks post-op | -1.17 ± 1.39 | -4.92 – 2.59 |
|  | Baseline – 12 weeks post-op | -0.51 ± 1.19 | -3.73 – 2.72 |
|  | Pre-op – 12 weeks post-op | -0.25 ± 1.26 | -3.64 – 3.15 |
| Shoulder extension | Baseline – pre-op | 1.55 ± 2.19 | -4.35 – 7.47 |
|  | Pre-op – 6 weeks post-op | -0.71 ± 2.69 | -7.98 – 6.57 |
|  | 6 weeks post-op - 12 weeks post-op | -1.66 ± 2.73 | -9.05 – 5.72 |
|  | Baseline – 6 weeks post-op | 0.86 ± 2.61 | -6.21 – 7.92 |
|  | Baseline – 12 weeks post-op | -0.81 ± 2.25 | -6.90 – 5.29 |
|  | Pre-op – 12 weeks post-op | -2.37 ± 2.35 | -8.72 – 3.98 |
| Shoulder abduction | Baseline – pre-op | 0.11 ± 1.29 | -3.37 – 3.60 |
|  | Pre-op – 6 weeks post-op | -1.56 ±1.62 | -5.97 – 2.78 |
|  | 6 weeks post-op - 12 weeks post-op | 0.51 ± 1.66 | -3.95 – 4.97 |
|  | Baseline – 6 weeks post-op | -1.48 ± 1.57 | -5.73 – 2.76 |
|  | Baseline – 12 weeks post-op | -0.97 ± 1.35 | -4.63 – 2.69 |
|  | Pre-op – 12 weeks post-op | -1.08 ± 1.42 | -4.90 – 2.74 |
| **Shoulder Range of Motion (°)** |  |  |  |
| Right Flexion | Baseline – pre-op | 3.61 ± 4.64 | -8.86 – 16.09 |
|  | Pre-op – 6 weeks post-op | -23.96 ± 5.43 | -38.54 – -9.38 |
|  | 6 weeks post-op - 12 weeks post-op | 8.39 ± 5.35 | -6.00 – 22.78 |
|  | Baseline – 6 weeks post-op | -20.34 ± 5.08 | -33.99 - -6.695 |
|  | Baseline – 12 weeks post-op | -11.95 ± 4.78 | -24.80 – 0.89 |
|  | Pre-op – 12 weeks post-op | -15.57 ± 5.06 | -29.16 - -1.97 |
| Left Flexion | Baseline – pre-op | 0.22 ± 3.44 | -9.05 – 9.50 |
|  | Pre-op – 6 weeks post-op | -5.49 ± 3.99 | -16.24 – 5.27 |
|  | 6 weeks post-op - 12 weeks post-op | 3.17 ± 3.86 | -7.24 – 13.58 |
|  | Baseline – 6 weeks post-op | -5.26 ± 3.67 | -15.16 – 4.64 |
|  | Baseline – 12 weeks post-op | -2.09 ± 3.45 | -11.39 – 7.21 |
|  | Pre-op – 12 weeks post-op | -2.31 ± 3.76 | -12.44 – 7.81 |
| Right Extension | Baseline – pre-op | 1.38 ± 2.78 | -6.12 – 8.87 |
|  | Pre-op – 6 weeks post-op | -5.63 ± 3.19 | -14.24 – 2.97 |
|  | 6 weeks post-op - 12 weeks post-op | 5.34 ± 3.10 | -3.03 – 13.70 |
|  | Baseline – 6 weeks post-op | -4.26 ± 2.78 | -12.27 – 3.75 |
|  | Baseline – 12 weeks post-op | 1.08 ± 2.80 | -6.47 – 8.63 |
|  | Pre-op – 12 weeks post-op | -0.30 ± 2.96 | -8.27 – 7.68 |
| Left Extension | Baseline – pre-op | - 1. 3.20 | -7.26 – 9.65 |
|  | Pre-op – 6 weeks post-op | 0.26 ± 3.62 | -9.49 – 10.01 |
|  | 6 weeks post-op - 12 weeks post-op | 2.46 ± 3.47 | -6.92 – 11.84 |
|  | Baseline – 6 weeks post-op | 1.28 ± 3.20 | -7.70 – 10.25 |
|  | Baseline – 12 weeks post-op | 3.74 ± 3.13 | -4.71 – 12.18 |
|  | Pre-op – 12 weeks post-op | 2.72 ± 3.41 | -6.47 – 11.91 |
| Right Abduction | Baseline – pre-op | 6.78 ± 6.04 | -9.49 – 23.04 |
|  | Pre-op – 6 weeks post-op | -19.78 ± 7.07 | -38.75 – -0.81 |
|  | 6 weeks post-op - 12 weeks post-op | 9.03 ± 6.97 | -9.72 – 27.79 |
|  | Baseline – 6 weeks post-op | -13.01 ± 6.62 | -30.79 – 4.77 |
|  | Baseline – 12 weeks post-op | -3.97 ± 6.23 | -20.71 – 12.76 |
|  | Pre-op – 12 weeks post-op | -10.75 ± 6.59 | -28.46 – 6.96 |
| Left Abduction | Baseline – pre-op | 0.03 ± 3.85 | -10.37 – 10.43 |
|  | Pre-op – 6 weeks post-op | -3.96 ± 4.47 | -16.01 – 8.10 |
|  | 6 weeks post-op - 12 weeks post-op | 5.79 ± 4.32 | -5.87 – 17.46 |
|  | Baseline – 6 weeks post-op | -3.92 ± 4.11 | -15.02 – 7.17 |
|  | Baseline – 12 weeks post-op | 1.87 ± 3.87 | -8.56 – 12.29 |
|  | Pre-op – 12 weeks post-op | 1.84 ± 4.21 | -9.51 – 13.19 |
| Right Internal Rotation | Baseline – pre-op | 7.01 ± 4.28 | -4.55 – 18.57 |
|  | Pre-op – 6 weeks post-op | -8.58 ± 5.11 | -22.34 – 5.17 |
|  | 6 weeks post-op - 12 weeks post-op | 7.51 ± 5.10 | -6.28 – 21.29 |
|  | Baseline – 6 weeks post-op | -1.58 ± 4.75 | -14.39 – 11.24 |
|  | Baseline – 12 weeks post-op | 5.93 ± 4.59 | -6.45 – 18.31 |
|  | Pre-op – 12 weeks post-op | -1.08 ± 4.87 | -14.23 – 12.08 |
| Left Internal Rotation | Baseline – pre-op | 4.67 ± 3.64 | -5.13 – 14.48 |
|  | Pre-op – 6 weeks post-op | -7.70 ± 4.21 | -19.04 – 3.64 |
|  | 6 weeks post-op - 12 weeks post-op | 10.64 ± 4.08 | -0.36 – 21.64 |
|  | Baseline – 6 weeks post-op | -3.03 ± 3.88 | -13.48 – 7.43 |
|  | Baseline – 12 weeks post-op | 7.61 ± 3.65 | -2.22 – 17.44 |
|  | Pre-op – 12 weeks post-op | 2.94 ± 3.97 | -7.75 – 13.63 |
| Right External rotation | Baseline – pre-op | 3.01 ± 3.98 | -7.75 – 13.77 |
|  | Pre-op – 6 weeks post-op | -6.39 ± 4.80 | -19.35 – 6.58 |
|  | 6 weeks post-op - 12 weeks post-op | -3.18 ± 4.68 | -15.83 – 9.48 |
|  | Baseline – 6 weeks post-op | -3.38 ± 4.42 | -15.32 – 8.56 |
|  | Baseline – 12 weeks post-op | -6.55 ± 4.12 | -17.69 – 4.59 |
|  | Pre-op – 12 weeks post-op | -9.56 ± 4.49 | -21.68 – 2.56 |
| Left External Rotation | Baseline – pre-op | 2.44 ± 2.84 | -5.24 – 10.11 |
|  | Pre-op – 6 weeks post-op | -4.47 ± 3.31 | -13.39 – 4.45 |
|  | 6 weeks post-op - 12 weeks post-op | -3.85 ± 3.19 | -12.45 – 4.76 |
|  | Baseline – 6 weeks post-op | -2.03 ± 3.04 | -10.23 – 6.16 |
|  | Baseline – 12 weeks post-op | -5.88 ± 2.85 | -13.58 – 1.82 |
|  | Pre-op – 12 weeks post-op | -8.32 ± 3.11 | -16.71 – 0.07 |
| **Lymphedema (cm)** |  |  |  |
| Right MCP joints | Baseline – pre-op | -0.46 ± 0.19 | -0.99 – 0.06 |
|  | Pre-op – 6 weeks post-op | 0.14 ± 0.23 | -0.48 – 0.76 |
|  | 6 weeks post-op - 12 weeks post-op | -0.12 ± 0.22 | -0.70 – 0.47 |
|  | Baseline – 6 weeks post-op | -0.33 ± 0.21 | -0.88 – 0.23 |
|  | Baseline – 12 weeks post-op | -0.44 ± 0.20 | -0.97 – 0.08 |
|  | Pre-op – 12 weeks post-op | 0.02 ± 0.21 | -0.55 – 0.59 |
| Left MCP joints | Baseline – pre-op | 0.15 ± 0.18 | -0.33 – 0.63 |
|  | Pre-op – 6 weeks post-op | -0.16 ± 0.21 | -0.72 – 0.41 |
|  | 6 weeks post-op - 12 weeks post-op | 0.09 ± 0.20 | -0.44 – 0.63 |
|  | Baseline – 6 weeks post-op | -0.01 ± 0.18 | -0.52 – 0.50 |
|  | Baseline – 12 weeks post-op | 0.08 ± 0.18 | -0.40 – 0.57 |
|  | Pre-op – 12 weeks post-op | -0.07 ± 0.19 | -0.59 – 0.46 |
| Right Wrist | Baseline – pre-op | -0.23 ± 0.17 | -0.68 – 0.23 |
|  | Pre-op – 6 weeks post-op | 0.15 ± 0.20 | -0.39 – 0.69 |
|  | 6 weeks post-op - 12 weeks post-op | -0.04 ± 0.19 | -0.55 – 0.47 |
|  | Baseline – 6 weeks post-op | -0.08 ± 0.18 | -0.57 – 0.41 |
|  | Baseline – 12 weeks post-op | -0.12 ± 0.17 | -0.58 – 0.34 |
|  | Pre-op – 12 weeks post-op | 0.11 ± 0.19 | -0.39 – 0.61 |
| Left Wrist | Baseline – pre-op | -0.15 ± 0.14 | -0.52 – 0.22 |
|  | Pre-op – 6 weeks post-op | 0.01 ± 0.16 | -0.43 – 0.45 |
|  | 6 weeks post-op - 12 weeks post-op | 0.24 ± 0.15 | -0.17 – 0.65 |
|  | Baseline – 6 weeks post-op | -0.14 ± 0.15 | -0.54 – 0.25 |
|  | Baseline – 12 weeks post-op | 0.10 ± 0.14 | -0.27 – 0.47 |
|  | Pre-op – 12 weeks post-op | 0.25 ± 0.15 | -0.16 – 0.65 |
| Right 10 cm distal to lateral epicondyle | Baseline – pre-op | -0.03 ± 0.26 | -0.72 – 0.67 |
|  | Pre-op – 6 weeks post-op | -0.03 ± 0.31 | -0.86 – 0.80 |
|  | 6 weeks post-op - 12 weeks post-op | -0.18 ± 0.29 | -0.97 – 0.60 |
|  | Baseline – 6 weeks post-op | -0.06 ± 0.28 | -0.80 – 0.69 |
|  | Baseline – 12 weeks post-op | -0.24 ± 0.26 | -0.94 – 0.46 |
|  | Pre-op – 12 weeks post-op | -0.21 ± 0.28 | -0.98 – 0.55 |
| Left 10 cm distal to lateral epicondyle | Baseline – pre-op | -0.19 ± 0.28 | -0.93 – 0.56 |
|  | Pre-op – 6 weeks post-op | 0.28 ± 0.33 | -0.60 – 1.16 |
|  | 6 weeks post-op - 12 weeks post-op | 0.22 ± 0.31 | -1.05 – 0.62 |
|  | Baseline – 6 weeks post-op | 0.10 ± 0.30 | -0.70 – 0.89 |
|  | Baseline – 12 weeks post-op | -0.12 ± 0.28 | -0.87 – 0.63 |
|  | Pre-op – 12 weeks post-op | 0.07 ± 0.30 | -0.75 – 0.88 |
| Right 15 cm proximal from lateral epicondyles | Baseline – pre-op | -0.66 ± 0.53 | -2.09 – 0.77 |
|  | Pre-op – 6 weeks post-op | 0.19 ± 0.63 | -1.50 – 1.89 |
|  | 6 weeks post-op - 12 weeks post-op | 0.20 ± 0.59 | -1.41 – 1.80 |
|  | Baseline – 6 weeks post-op | -0.47 ± 0.57 | -2.00 – 1.06 |
|  | Baseline – 12 weeks post-op | -0.27 ± 0.53 | -1.71 – 1.17 |
|  | Pre-op – 12 weeks post-op | 0.39 ± 0.58 | -1.17 – 1.96 |
| Left 15 cm proximal from lateral epicondyles | Baseline – pre-op | -0.42 ± 0.40 | -1.05 – 0.66 |
|  | Pre-op – 6 weeks post-op | -0.02 ± 0.47 | -1.30 – 1.26 |
|  | 6 weeks post-op - 12 weeks post-op | 0.13 ± 0.45 | -1.07 – 1.34 |
|  | Baseline – 6 weeks post-op | -0.44 ± 0.43 | -1.59 – 0.71 |
|  | Baseline – 12 weeks post-op | -0.31 ± 0.40 | -1.39 – 0.78 |
|  | Pre-op – 12 weeks post-op | 0.11 ± 0.44 | -1.07 – 1.29 |

Supplemental Table 2: Mean Timepoint Differences for Participant Reported Outcomes (n=22)

| **Outcome** | **Contrast** | **Δ ± SE** | **95% CI** |
| --- | --- | --- | --- |
| **GLTEQ** | Baseline – pre-op | 15.11 ± 5.83 | -0.72 – 30.95 |
|  | Pre-op – 6 weeks post-op | -16.21 ± 6.70 | -34.41 – 1.99 |
|  | 6 weeks post-op - 12 weeks post-op | 12.09 ± 6.32 | -5.14 – 29.32 |
|  | Baseline – 6 weeks post-op | -1.10 ± 6.08 | -17.63 – 15.44 |
|  | Baseline – 12 weeks post-op | 10.99 ± 5.87 | -4.96 – 26.94 |
|  | Pre-op – 12 weeks post-op | -4.12 ± 6.36 | -21.41 – 13.17 |
| **WHODAS: Average Disability Score** | Baseline – pre-op | -0.76 ± 2.07 | -6.37 – 4.85 |
|  | Pre-op – 6 weeks post-op | 4.04 ± 2.42 | -2.54 – 10.61 |
|  | 6 weeks post-op - 12 weeks post-op | -3.06 ± 2.36 | -9.46 – 3.35 |
|  | Baseline – 6 weeks post-op | 3.27 ± 2.22 | -2.76 – 9.30 |
|  | Baseline – 12 weeks post-op | 0.22 ± 2.15 | -5.60 – 6.04 |
|  | Pre-op – 12 weeks post-op | 0.98 ± 2.30 | -5.26 – 7.22 |
| **FACT-F** | Baseline – pre-op | 1.84 ± 3.23 | -6.90 – 10.57 |
|  | Pre-op – 6 weeks post-op | -3.73 ± 3.78 | -13.9 – 6.47 |
|  | 6 weeks post-op - 12 weeks post-op | -2.74 ± 3.71 | -12.8 – 7.31 |
|  | Baseline – 6 weeks post-op | -1.89 ± 3.46 | -11.3 – 7.48 |
|  | Baseline – 12 weeks post-op | -4.63 ± 3.34 | -13.7 – 4.41 |
|  | Pre-op – 12 weeks post-op | -6.47 ± 3.60 | -16.2 – 3.27 |
| **BPI: Severity** | Baseline – pre-op | 0.40 ± 0.36 | -0.58 – 1.38 |
|  | Pre-op – 6 weeks post-op | -0.31 ± 0.42 | -1.46 – 0.84 |
|  | 6 weeks post-op - 12 weeks post-op | 0.10 ± 0.41 | -1.02 – 1.22 |
|  | Baseline – 6 weeks post-op | 0.09 ± 0.39 | -0.96 – 0.14 |
|  | Baseline – 12 weeks post-op | 0.19 ± 0.38 | -0.82 – 1.21 |
|  | Pre-op – 12 weeks post-op | 0.21 ± 0.40 | -1.30 – 0.88 |
| **BPI: Interference** | Baseline – pre-op | 0.15 ± 0.46 | -1.10 – 1.40 |
|  | Pre-op – 6 weeks post-op | 0.69 ± 0.54 | -0.76 – 2.15 |
|  | 6 weeks post-op - 12 weeks post-op | -0.64 ± 0.53 | -2.07 – 0.78 |
|  | Baseline – 6 weeks post-op | 0.85 ± 0.49 | -0.49 – 2.18 |
|  | Baseline – 12 weeks post-op | 0.20 ± 0.48 | -1.09 – 1.50 |
|  | Pre-op – 12 weeks post-op | 0.05 ± 0.51 | -1.34 – 1.44 |
| **DASH** | Baseline – pre-op | 2.99 ± 4.34 | -8.79 – 14.76 |
|  | Pre-op – 6 weeks post-op | 16.18 ± 4.96 | 2.74 – 29.63 |
|  | 6 weeks post-op - 12 weeks post-op | -7.20 ± 4.79 | -20.22 – 5.82 |
|  | Baseline – 6 weeks post-op | 19.17 ± 4.51 | 6.96 – 31.38 |
|  | Baseline – 12 weeks post-op | 11.97 ± 4.35 | 0.18 – 23.76 |
|  | Pre-op – 12 weeks post-op | 8.99 ± 4.72 | -20.22 – 5.82 |
| **SF-36: PCS** | Baseline – pre-op | -1.76 ± 2.10 | -7.42 – 3.91 |
|  | Pre-op – 6 weeks post-op | -3.23 ± 2.45 | -9.82 – 3.37 |
|  | 6 weeks post-op - 12 weeks post-op | -0.92 ± 2.42 | -7.46 – 5.63 |
|  | Baseline – 6 weeks post-op | -4.98 ± 2.25 | -11.05 – 1.08 |
|  | Baseline – 12 weeks post-op | -5.90 ± 2.17 | -11.75 - -0.05 |
|  | Pre-op – 12 weeks post-op | -4.14 ± 2.34 | -10.47 – 2.18 |
| **SF-36: MCS** | Baseline – pre-op | -1.79 ± 1.90 | -6.99 – 3.40 |
|  | Pre-op – 6 weeks post-op | 4.36 ± 2.25 | -1.72 – 10.44 |
|  | 6 weeks post-op - 12 weeks post-op | -0.67 ± 2.20 | -6.63 – 5.29 |
|  | Baseline – 6 weeks post-op | 2.57 ± 2.06 | -3.01 – 8.15 |
|  | Baseline – 12 weeks post-op | 1.90 ± 1.99 | -3.49 – 7.28 |
|  | Pre-op – 12 weeks post-op | 3.69 ± 2.14 | -2.10 – 9.48 |

GLTEQ: Godin Leisure Time Exercise Questionnaire; WHODAS: World Health Organization Disability Assessment Schedule; FACT-F: Functional Assessment of Cancer Therapy – Fatigue; BPI: Brief Pain Inventory; DASH: Disabilities of Arm, Shoulder, and Hand; SF-36 PCS: 36 Item Short Form Survey Physical Component Score; SF-36 MCS: 36 Item Short Form Survey Mental Component Score
